# Case Report: When inotropy backfires—dobutamine-associated eosinophilic myocarditis

**DOI:** 10.3389/fcvm.2026.1781601

**Published:** 2026-04-30

**Authors:** Abdullah Aljudaibi, Kristi Dutta, Sahitya Allam, Jose-Alejandro Almario, Lo Tamburro, Lynn Dees, Erik Sorensen, Bartley P. Griffith, Gautam Ramani, Manjula Ananthram

**Affiliations:** 1 Department of Medicine, University of Maryland School of Medicine, Baltimore, MD, United States; 2 Department of Cardiovascular Medicine, Cooper University Hospital, Camden, NJ, United States; 3 Division of Cardiovascular Medicine, University of Pittsburgh Medical Center—Harrisburg, Harrisburg, PA, United States; 4 Department of Pathology, University of Maryland School of Medicine, Baltimore, MD, United States; 5 Division of Cardiovascular Medicine, University of Maryland School of Medicine, Baltimore, MD, United States; 6 Division of Perioperative Services, Department of Surgery, University of Maryland Medical Center, Baltimore, MD, United States; 7 Division of Cardiothoracic Surgery, Department of Surgery, University of Maryland School of Medicine, Baltimore, MD, United States

**Keywords:** acute heart failure, cardiac assist devices, chronic heart failure, eosinophilia, inotropes, left ventricle, myocarditis, right ventricle

## Abstract

Eosinophilic myocarditis (EM) is a rare form of myocarditis that is often triggered by drug hypersensitivity reactions. We describe the case of a 55-year-old man who presented with cardiogenic shock that rapidly progressed despite dual inotropic therapy, ultimately requiring left ventricular assist device implantation. A left ventricular apical core biopsy demonstrated eosinophilic infiltration consistent with EM. After comprehensive evaluation of the possible etiologies, dobutamine was identified as the most likely offending agent. Following discontinuation of dobutamine and initiation of corticosteroid therapy, ventricular function and hemodynamics improved, supporting a diagnosis of dobutamine-associated EM. Clinicians should consider drug-induced EM in patients with refractory cardiogenic shock receiving dobutamine. Alternative inotropic agents such as milrinone or epinephrine should be considered.

## History of presentation

A 55-year-old man with a history of dilated non-ischemic cardiomyopathy (NICM) presented to the heart failure clinic with three weeks of worsening exertional dyspnea, orthopnea, and lower extremity edema despite recent medication adjustments. He denied antecedent viral syndrome, chest pain, or new rash. He was referred to the emergency department for evaluation. On admission, his weight was 129.7 kg (286 lb) and height was 1.82 m (6 ft 1 in.), with a BMI of 39.1 kg/m^2^. Initial vitals demonstrated mild tachycardia (102 beats/min) and tachypnea (24 breaths/min). Mean arterial pressure was maintained in the 80–90 mmHg range. Physical examination revealed conversational dyspnea, jugular venous distension, weight gain of 3.5 kg from baseline, and severe pitting edema in the lower extremities, which were cool to touch.

An electrocardiogram (EKG) showed sinus tachycardia. Laboratory results were notable for creatinine at baseline (1.67 mg/dL), elevated N-terminal-pro brain natriuretic peptide (NT-proBNP) (2,500 pg/mL), and elevated lactate (2.5 mmol/L). Troponin I levels were flat at 0.05 ng/mL on three measurements. Chest X-ray revealed cardiomegaly.

An urgent transthoracic echocardiogram (TTE) revealed a dilated left ventricle with an ejection fraction (EF) of 15%–20%, a 7 mm × 5 mm echodensity suggestive of a left ventricular (LV) thrombus, and mild to moderately decreased right ventricular (RV) function. His most recent TTE 3 months earlier had demonstrated similar biventricular function.

He was treated with aggressive diuresis, bilevel positive airway pressure (BiPAP), and low-dose dobutamine (2.5 μg/kg/min). A summary of the patient's clinical course is provided in Table 1 for reference.

**Table 1:** Visual summary of case.

Hospital day (HD)	Key events/procedures	Inotropes/vasopressors	Labs	Respiratory/oxygenation
HD 1	Presentation with progressive NYHA IV symptoms; cardiogenic shock	Started dobutamine 2.5 μg/kg/min	AEC 0.0 K/µL (0.5%)	BiPAP
HD 2	TTE: severe LV dysfunction; moderate RV dysfunction. RHC: elevated filling pressures, low CI; Swan–Ganz left in place	Dobutamine increased to 5 μg/kg/min (max)	—	BiPAP
HD 3–7	Persistent shock; fevers, hemoptysis, hypoxia → bronchoscopy (mild hemosiderosis); infectious/autoimmune workup negative	Milrinone started 48 h after dobutamine: 0.25 to 0.375 μg/kg/min Vancomycin and piperacillin/tazobactam started	AEC 0.3 K/µL (3.8%); CRP 15.7 mg/dL	BiPAP → weaned to nasal cannula
HD 8	IABP placed for mechanical circulatory support	Dobutamine 5 μg/kg/min + milrinone 0.25–0.375 μg/kg/min	—	—
HD 10	Pre-operative optimization	Dobutamine discontinued (end of exposure)	—	—
HD 11	HeartMate 3 LVAD implantation + LV apical core biopsy; intraoperative worsening RV function	Milrinone continued (postoperative support begins) Vancomycin and piperacillin/tazobactam discontinued	—	—
Post-op period	Persistent severe biventricular dysfunction; CRRT required	Epinephrine 0.05–0.06 μg/kg/min (transient increase 0.4–0.6); Norepinephrine 0.06 μg/kg/min (intermittent); Milrinone continued	—	Weaned from BiPAP → nasal cannula; SpO_2_ 95%–99%
HD 21–22	Biopsy resulted: eosinophilic myocarditis; corticosteroids initiated	Milrinone continued		Nasal cannula
Post-steroids (3–5 days)	Clinical stabilization; improvement in RV function on TTE	—	AEC 0.0 K/µL; CRP 1.9 mg/dL	Nasal cannula
HD 39	Repeat TTE: mild–moderate RV dysfunction; milrinone discontinued	Milrinone discontinued	—	—

BiPAP, bilevel positive airway pressure; TTE, transthoracic echocardiogram; LVEF, left ventricular ejection fraction; RHC, right heart catheterization; IABP, intra-aortic balloon pump; LVAD, left ventricular assist device; CRRT, continuous renal replacement therapy; GDMT, guideline-directed medical therapy; AEC, absolute eosinophil count; CRP, C-reactive protein.

## Past medical history

The patient has a history of dilated NICM with multiple recent hospital admissions for acute decompensated heart failure (ADHF). These prior admissions were managed with intravenous diuresis, short-term inotropy with dobutamine, and optimization of guideline-directed medical therapy (GDMT), which resulted in temporary clinical improvement. During these previous admissions, there was no concern for peripheral eosinophilia or myocarditis. He had no history suggestive of hypereosinophilic syndrome.

The patient's other medical conditions included severe mitral regurgitation, stage 3a chronic kidney disease, type 2 diabetes complicated by peripheral neuropathy, and hyperlipidemia. His GDMT consisted of eplerenone 50 mg daily, empagliflozin 10 mg daily, torsemide 40 mg daily, and sacubitril-valsartan 24–26 mg twice daily.

## Differential diagnosis

At the time of hospital admission, the most pressing diagnosis was ADHF complicated by Society for Cardiovascular Angiography and Interventions (SCAI) Stage C cardiogenic shock. Progressive mitral regurgitation, pulmonary hypertension, and pneumonia were also considered due to his profound dyspnea. Myocardial ischemia and infarction were lower on the differential due to a lack of ST/T wave changes on EKG, troponins with significant change, or regional wall motion abnormalities on TTE. The patient also had a recent normal outpatient nuclear stress test. Myocarditis and pericarditis were also lower on the differential at presentation due to a lack of characteristic EKG changes, regional wall motion abnormalities or pericardial effusion, or significantly elevated troponins.

## Investigations

An intravenous heparin drip was initiated to treat the LV apical thrombus. After limited improvement with empiric diuresis and low-dose dobutamine, the patient underwent right heart catheterization (RHC) on day 2 of hospitalization, which revealed elevated biventricular filling pressures with prominent V waves indicating severe mitral regurgitation, as well as a reduced cardiac output and index (CI). A summary of the hemodynamic measurements from the RHC and intracardiac monitoring is provided in Table 2. A Swan–Ganz catheter was left in place to allow for hemodynamic tailored therapy. A summary of the patient's hemodynamics, inotropes, and mechanical circulatory support (MCS) requirements over the subsequent days is provided in Table 1. Over the next 48 h, he required up-titration of dobutamine to 5 μg/kg/min and addition of milrinone at 0.25–0.375 μg/kg/min.

**Table 2:** Measurements from RHC and Swan–Ganz catheter.

Normal reference ranges	Right atrial pressure or CVP (mmHg)	Right ventricle pressure (mmHg)	Pulmonary artery (mean) (mmHg)	PCWP (mmHg)	FICK CO/CI (mmHg)	SVR/PVR^a^ (dyn·s/cm^5^)	SvO2 (%)
Normal	0–5	25/5	25/10	6–12	4–8 L/min or 2.5–4.0 L/min/m^2^	800–1,200	65%–80%
RHC day 2	15	78/14	78/34 (49)	28	3.85/1.56	1,315/277	48%

Intracardiac monitoring	CVP	PA (mean)	FICK CO/CI	SvO2
Day 3	33	77/46 (56)	3.3/1.3	38%
Day 6	15	54/25 (35)	4.9/1.91	50%
Day 7	15	64/33 (43)	4.2/1.66	37.6%
RHC day 8, IABP	16	69/28 (42)	4.35/1.75	45%
Day 10	22	75/25 (42)	4.7/1.9	47%
Day 11 (post LVAD)	16	33/17 (23)	4.9/2.2^b^	52%
Day 17	15	46/25 (34)	NA/3.4	66%
Day 18	8	36/24 (29)	NA/3	57%

MAP, mean arterial pressure; CVP, central venous pressure; CO, cardiac output; mPAP, mean pulmonary artery pressure; FICK CO/CI, fick-derived cardiac output/cardiac index; PCWP, pulmonary capillary wedge pressure, the multiplication by 80 standardizes the units.

The multiplier of 80 converts the result to dynes·s/cm^5^, the standard unit for vascular resistance.

^a^ Standard formulas were used to calculate vascular resistance in the cardiovascular system. In particular, we applied the following:

Systemic vascular resistance $\mathrm{SVR}=\frac{\left(\mathrm{MAP}-\mathrm{CVP}\right)}{\mathrm{CO}}\times 80$.

Pulmonary vascular resistance $\mathrm{PVR}=\frac{\left(\mathrm{mPAP}-\mathrm{PCWP}\right)}{\mathrm{CO}}\times 80$.

^b^ After day 11 following LVAD implantation, the cardiac output readings from Swan–Ganz catheter were unreliable due to the continuous flow dynamics of the device. Therefore, measurements derived from the cardiac output such as the SVR and PVR were not calculated.

**Figure 1:**
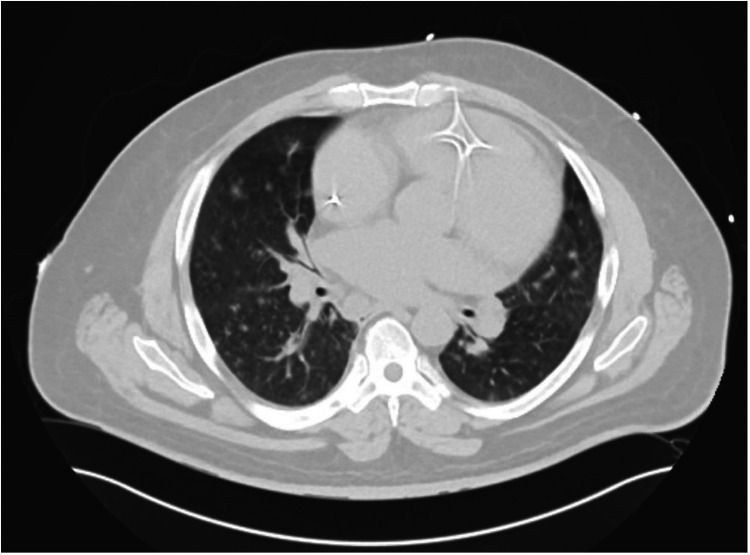
Computerized tomography without contrast of the chest showing innumerable bilateral pulmonary nodules and small ground-glass opacities compatible with pneumonia, prominent septal markings likely reflecting associated pulmonary edema, and marked cardiac chamber enlargement.

His course was complicated by atrial fibrillation with rapid ventricular response that was responsive to an amiodarone infusion (delivered at rates up to 1 mg/min between days 7 and 16). Concurrently, he developed intermittent fevers, hemoptysis, and hypoxia, raising concern for alternative processes such as pulmonary emboli, vasculitis, or pneumonia. He was started on broad-spectrum antibiotics including vancomycin (dosed by pharmacy using trough levels) and piperacillin/tazobactam (3.375 mg every 6 h), which were continued for 4 days (days 7–11). CT chest showed diffuse pulmonary nodules concerning for an infectious or autoimmune process (Figure 1). Bronchoscopy showed mild hemosiderosis, likely from chronic volume overload. Infectious and autoimmune workups were unrevealing, apart from a mildly positive anti-nuclear antibody titer (1:80).

On day 8, an intra-aortic balloon pump was placed for temporary MCS in addition to dual inotropes. Despite deteriorating shock, the patient did not required intubation. Dobutamine was discontinued on day 10. Ultimately, the patient underwent urgent HeartMate 3 left ventricular assist device (LVAD) implantation on day 11, during which an LV apical core biopsy was performed intraoperatively.

## Management

**Figure 2:**
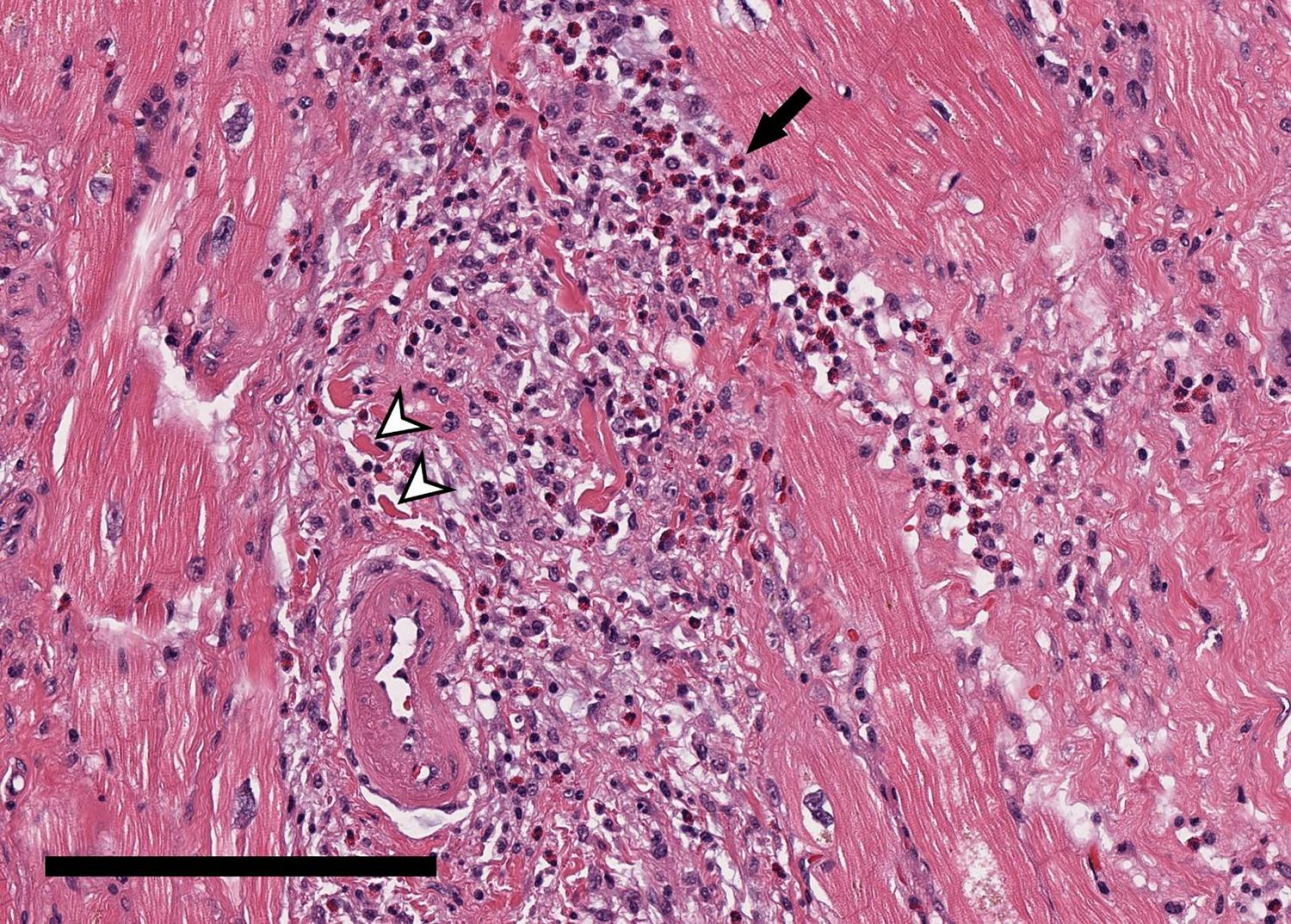
20× magnification view of the apical myocardium showing extensive interstitial and perivascular mixed inflammatory cell infiltrates consisting of many eosinophils (arrow) with histiocytes, lymphocytes and plasma cells with focal myocyte necrosis (arrowhead), consistent with eosinophilic (hypersensitivity) myocarditis. (H&E stain; scale bar: 200 μm).

During LVAD implantation, moderate to severe RV dysfunction was noted on intraoperative transesophageal echocardiography, which was more pronounced than on initial TTE (Supplementary Videos 1 and 2). Inotropes and pulmonary vasodilators were continued postoperatively [milrinone (0.25–0.375 μg/kg/min), epinephrine (0.05–0.06 μg/kg/min with transient escalation to 0.4–0.6 μg/kg/min in the immediate postoperative period), norepinephrine (0.06 μg/kg/min), inhaled epoprostenol, and nitric oxide], but dobutamine was not resumed. TTE on day 15 showed persistent severe biventricular dysfunction. Cardiorenal syndrome worsened, resulting in the need for continuous renal replacement therapy (CRRT). On day 21, the LV apical core biopsy was reported as eosinophilic myocarditis (EM) (Figure 2).

Based on the association between prolonged dobutamine exposure and biopsy-proven eosinophilic myocarditis, dobutamine was suspected to be the offending agent. Alternative etiologies could not be definitively excluded, including milrinone, amiodarone, antibiotics, and systemic inflammation. However, laboratory serologies were most suggestive of the dobutamine hypothesis (Table 1). On admission, the absolute eosinophil count was 0.0 K/μL (0.5%). After 1 week of continuous dobutamine therapy (day 7), eosinophils increased to 3.8% (absolute count 0.3 K/μL). Amiodarone and antibiotics had only just been initiated on day 7. On the morning of LVAD implantation (day 11), C-reactive protein was elevated at 15.7 mg/dL. Following initiation of corticosteroid therapy (days 22–27), eosinophils declined to 0.0% (absolute count 0.0 K/μL), and C-reactive protein (CRP) down-trended to 1.9 mg/dL. Milrinone was continued throughout steroid therapy.

## Outcome and follow-up

Intravenous methylprednisolone 65 mg daily (approximately 0.5 mg/kg/day) was initiated for 2 days, followed by an oral prednisone taper. Five days after starting steroids (day 27), repeat TTE showed improved RV function.

The patient was weaned off all inotropes except milrinone, which supported renal recovery. CRRT was discontinued shortly after. On day 39, TTE showed mild RV dysfunction and milrinone was stopped. The patient was discharged on oral diuretics, GDMT, and stable LVAD settings.

## Discussion

EM is a rare form of myocarditis that is defined by eosinophilic infiltration of the myocardium. It is often associated with hypersensitivity reactions and eosinophilic disorders, such as parasitic infections and eosinophilic granulomatosis with polyangiitis. It has a wide range of clinical manifestations, including chest pain, arrhythmias, heart failure, and cardiogenic shock (1).

In drug-induced EM, injury arises from a T-cell-mediated immune response to a drug or its metabolites (2). Features such as eosinophilia, fever, rash, and new or worsening heart failure may suggest EM, but some patients are asymptomatic (3). Common medications associated with EM include antibiotics—such as penicillin, cephalosporins, and sulfonamides—as well as neuropsychiatric agents like clozapine (1).

Dobutamine, a commonly used inotropic agent, has been identified as a rare cause of EM. Some formulations contain sodium bisulfite, a known allergen that can trigger hypersensitivity (1). The specific formulation administered in this case could not be verified, but the contribution of sodium bisulfite remains speculative. The incidence of hypersensitivity myocarditis was found to be 2%–7% in patients awaiting cardiac transplantation who were on multiple medications, including dobutamine (3). A recent cohort study reported that among patients on continuous inotropes awaiting transplant, 14% of those receiving dobutamine developed EM, whereas none who received milrinone developed EM (4). These findings suggest that extended dobutamine use could be a trigger for EM and that milrinone may be a safer option in these situations.

Indicators of dobutamine-associated EM can include worsening heart failure days to weeks after starting dobutamine, sometimes associated with eosinophilia and fever that can mimic sepsis. However, infectious workups are typically negative, as in our case (1). A case by Raje et al. described a man with dilated cardiomyopathy, whose right ventricular failure worsened on dobutamine, with escalating leukocytosis and peripheral eosinophilia. His clinical condition necessitated mechanical support, and biopsy revealed a diagnosis of EM (5). In another case by Maaliki et al., a patient developed fever and eosinophilia one week after starting dobutamine, improving with cessation and steroid administration (6). In another case of a heart transplant candidate, dobutamine caused persistent fevers; ultimately, he defervesced when dobutamine was discontinued. Following the transplant, the explanted heart revealed EM with features consistent with an etiology of drug-induced myocarditis. Throughout his course, he had no peripheral eosinophilia (7). The variability in presentation of EM necessitates a low threshold to consider it as an etiology of clinical decompensation and to identify potential triggers.

The gold standard for diagnosing EM is an endomyocardial biopsy (EMB) (8). Peripheral eosinophilia is not required for the diagnosis of eosinophilic myocarditis and may be absent or mild, as in our case, necessitating histopathologic confirmation. Diagnosis is confirmed when biopsy reveals inflammatory cell infiltrate containing a variable amount of eosinophils and can be accompanied by myocyte necrosis when there is florid infiltration (1, 8). Because infiltration is typically patchy, EMB sensitivity is around 50%, requiring multiple samples or a repeat biopsy (8). EMB is invasive and does carry risk of trauma to the tissue; thus, as such, EM is frequently discovered incidentally during cardiac transplantation or device implantation (9). In retrospect, earlier consideration of EMB could have been debated in this case with refractory shock and unexplained inflammatory features. However, the initial clinical picture was confounded by suspected infection and respiratory findings, appropriately prioritizing stabilization and infectious evaluation before invasive myocardial sampling.

Cardiac magnetic resonance (CMR) can be used when EMB results are inconclusive. CMR can identify patterns of injury and inflammation that suggest myocarditis. Typical findings include myocardial edema on T2-weighted images and patchy, subendocardial late gadolinium enhancement (LGE). CMR is also useful for monitoring disease progression and response to therapy. It complements but does not replace biopsy, which is needed for definitive diagnosis (8). In this case, CMR was not performed due to hemodynamic instability and the need for mechanical circulatory support in the acute setting.

Cardiac biomarkers are often elevated in EM. Troponin and creatine kinase (CK-MB) reflect myocardial injury and may be mild in subacute cases or significantly elevated in fulminant cases. BNP levels often rise with worsening ventricular function, but these markers are not specific to myocarditis. Eosinophil cationic protein (ECP), released during eosinophil activation, is a possible marker of disease activity. Levels tend to fall with treatment and rise again with flares, though ECP is not yet widely used in clinical practice (8).

Treatment in drug-induced EM includes stopping the offending agent and starting high-dose corticosteroids. Reports demonstrate full recovery of cardiac function in many patients, even those with severe heart failure, when treatment is started early (1, 8). For dobutamine-induced EM, treatment often leads to excellent outcomes, but if not recognized early, fulminant cases can decompensate rapidly, carrying a mortality rate of 36%. Ultimately, some of these patients may require LVAD implantation or cardiac transplantation (10). To reduce relapse, steroids are tapered gradually or maintained at low doses for extended periods (1). Follow-up typically involves serial echocardiography to assess ventricular recovery. Repeat CMR or biopsy may be considered to confirm resolution (8).

Importantly, this case supports an association rather than proven causality. Alternative explanations were considered exhaustively in this patient, including natural trajectory of progressive heart failure, pulmonary emboli, systemic vasculitis, infectious etiologies, hypersensitivity myocarditis related to other agents (e.g., milrinone, amiodarone), perioperative drug exposures, and systemic inflammatory stress during critical illness. However, the inciting trigger remains unclear. The features of the clinical course that most strongly support a dobutamine-associated process include development of fever 1 week after initiating dobutamine, documented modest eosinophilia 10 days following dobutamine, and resolution of RV failure and shock with corticosteroids and dobutamine cessation. Ultimately, other etiologies were excluded with laboratory values, imaging findings, and exposure timelines (amiodarone and antibiotics were started after the onset of fever; cardiac function improved despite continuation of milrinone until day 39).

In summary, drug-induced EM is a potentially reversible cause of cardiac dysfunction. While dobutamine is not widely recognized as a trigger of EM, it should be considered in advanced heart failure patients who unexpectedly worsen despite standard therapies. Recognizing the signs of inflammation and eosinophilia early, promptly stopping the culprit drug, and starting steroids can lead to full recovery in many cases. EMB and CMR help guide diagnosis and treatment. Most patients have a favorable prognosis and recovery, but more research is needed to guide long-term follow-up and develop treatment protocols.

## Limitations

This case has several limitations. First, as a case report, causality cannot be definitively established. Although biopsy confirmed eosinophilic myocarditis, multiple concurrent exposures and acute critical illness may have contributed. Second, peripheral eosinophilia was modest, which limits reliance on laboratory trends alone. Third, CMR was not performed due to clinical instability and mechanical circulatory support. Therefore, the association with dobutamine should be interpreted cautiously within the broader clinical context.

## Take-home messages

Eosinophilic myocarditis is an under-recognized but serious complication of dobutamine. If suspected, the patient should be switched from inotropy to milrinone or epinephrine treatment, an endomyocardial biopsy should be considered, and high-dose steroids should be started early to improve outcomes.

Written informed consent was obtained from the patient for publication of this case report and associated images. Institutional review board approval was not required for a single-patient case report in accordance with institutional policy.

## Data Availability

The original contributions presented in the study are included in the article/Supplementary Material, further inquiries can be directed to the corresponding author.

## Supplementary material

Supplementary Video 1 Intraoperative TEE, pre-LVAD placement (on dobutamine 5 μg/kg/min and milrinone 0.3 μg/kg/min) showing significant RV dilation with persistent, severely reduced right ventricular function.

Supplementary Video 2 Intraoperative TEE, post-LVAD placement (epinephrine 0.05 μg/kg/min, norepinephrine 0.03 μg/kg/min, and milrinone 0.3 μg/kg/min) showing persistent, severely reduced right ventricular function.
